# The Role of MicroRNAs in Diabetic Complications—Special Emphasis on Wound Healing

**DOI:** 10.3390/genes5040926

**Published:** 2014-09-29

**Authors:** João Moura, Elisabet Børsheim, Eugenia Carvalho

**Affiliations:** 1 Center for Neuroscience and Cell Biology, University of Coimbra, Coimbra 3004-517, Portugal; E-Mail: jmouraalves@gmail.com; 2 Arkansas Children’s Nutrition Center, Little Rock, Arkansas, AR 72202, USA; E-Mail: EBorsheim@uams.edu; 3 Arkansas Children’s Hospital Research Institute, Little Rock, AR 72202, USA; 4 Department of Pediatrics, University of Arkansas for Medical Sciences, Little Rock, AR 72202, USA; 5 Department of Geriatrics, University of Arkansas for Medical Sciences, Little Rock, AR 72202, USA; 6 The Portuguese Diabetes Association (APDP), Lisbon 1250 203, Portugal

**Keywords:** microRNA, diabetes, macrovascular and microvascular complications, skin, wound healing, inflammation, vascular disease, diagnostic biomarkers, therapeutic targets

## Abstract

Overweight and obesity are major problems in today’s society, driving the prevalence of diabetes and its related complications. It is important to understand the molecular mechanisms underlying the chronic complications in diabetes in order to develop better therapeutic approaches for these conditions. Some of the most important complications include macrovascular abnormalities, e.g., heart disease and atherosclerosis, and microvascular abnormalities, e.g., retinopathy, nephropathy and neuropathy, in particular diabetic foot ulceration. The highly conserved endogenous small non-coding RNA molecules, the micro RNAs (miRNAs) have in recent years been found to be involved in a number of biological processes, including the pathogenesis of disease. Their main function is to regulate post-transcriptional gene expression by binding to their target messenger RNAs (mRNAs), leading to mRNA degradation, suppression of translation or even gene activation. These molecules are promising therapeutic targets and demonstrate great potential as diagnostic biomarkers for disease. This review aims to describe the most recent findings regarding the important roles of miRNAs in diabetes and its complications, with special attention given to the different phases of diabetic wound healing.

## 1. Diabetes and Its Complications

The prevalence of diabetes, a chronic metabolic disorder, has been increasing at alarming rates all over the world and is estimated to rise to 552 million adults by 2030 [1,2]. The associated increase in mortality and morbidity makes it one of the major health and socio-economic problems in our society [1,3]. The concurrent overweight and obesity epidemic, due to poor diet and lack of physical activity and interactions with genetic predisposition, greatly affect the prevalence of diabetes [1,2]. Type 2 diabetes mellitus (T2DM), in particular, which used to be predominantly a disease of adults, is now commonly observed at an early age in children and adolescents [1,3]. The increasing incidence of obesity in children and the resultant insulin resistance contributes to the increasing prevalence of T2DM in this population.

T2DM comprises approximately 90% of all diabetic patients and is characterized by hyperglycemia. This is mainly caused by lack of insulin, or loss of insulin sensitivity in the target organs in the presence of normal insulin secretion, or a combination of both. In contrast, type 1 diabetes is characterized by the absolute lack of insulin due to failure of the beta cell. Insulin resistance, which is the impaired ability of insulin to elicit its metabolic effects in the target tissues, particularly, in fat, liver and skeletal muscle, is one of the important causes of T2DM and cardiovascular disease [4,5]. Long-term hyperglycemia can lead to serious macrovascular and microvascular complications. Accelerated atherosclerosis and hypertension, common in diabetic patients, tend to cause macrovascular complications, such as cardiovascular deterioration and ultimately coronary heart disease and stroke [6,7]. On the other hand, microvascular events cause diabetic retinopathy [8,9], a leading cause of blindness in patients, and diabetic nephropathy [10,11], affecting the kidneys with disruption of glomeruli, tubules, vessels and the interstitium, causing impairment in renal function and ultimately leading to end-stage renal disease. In addition, microvascular disease can also cause diabetic neuropathy [3,12,13], affecting in particular the nervous system, ultimately leading to the development of chronic diabetic foot ulcers (DFU), among other complications.

The chronic inflammatory state characteristic of T2DM, leading to its severe chronic complications, can be delayed or even prevented by proper nutrition and regular physical exercise, at an early stage. However, the need to find better and more effective treatment solutions for these long-term complications in patients remains of importance. Several large clinical trials have been performed to address some of these questions [14,15,16]. The results coming from these large studies show that even though glucose-lowering treatment reduces the risk of cardiovascular diseases, the risks of macrovascular and microvascular complications still remain, and the development of new therapeutic strategies is needed. It has been suggested that the main reason these large studies have not been able to show any major impact on lowering the risk for diabetic complications, is that the interventions are implemented too late after the diagnosis of the disease. This is supported by the UKPDS and STENO-2 studies, where treatment of chronic hyperglycemia was undertaken at the early stages of the disease [17,18]. These interventions resulted in lower glycemia and long-term reduction of the risk of macrovascular and microvascular complications. However, most of the currently used therapies are not fully efficacious and therefore, there is an urgent need for a better understanding of the biomolecular mechanisms underlying T2DM development and its complications in order to identify better therapeutic targets.

The highly conserved endogenous small non-coding RNA molecules, micro RNAs (miRNAs or miR) are 18–25 nucleotides in length. They have recently been shown to be involved in the regulation of many key biological functions in both physiological and pathophysiological states, including the maintenance of cellular signaling and the regulation of entire pathways. When the tight miRNA regulation is altered it can lead to serious physiological abnormalities, including chronic diseases, such as diabetes and its complications [19,20,21,22]. Their main function is to regulate post-transcriptional gene expression by binding to their target messenger RNAs (mRNAs), leading to mRNA degradation or suppression of translation [19,20,21]. These molecules are promising therapeutic targets and demonstrate great potential as diagnostic biomarkers for the different diabetes complications. This review summarizes first the most recent findings regarding the important role of miRNA in the general pathology of diabetic complications, focusing toward the end, on the less studied, diabetic skin wound healing.

## 2. MiRNAs in Diabetes Complications

The extraordinary discovery of miRNAs by Ambros and co-workers, in 1993, gave new insights into the regulation of the genome, and further findings in recent years have impacted our understanding of gene regulation at the post-transcriptional level [23]. They are a novel class of non-coding RNAs that are expressed in all tissues and play major roles in human diseases, including diabetes [19,20,21]. More importantly, these miRNAs can also be found in the circulation, thus being available as biomarkers for monitoring of disease onset and progression. Moreover, the circulating miRNAs are reflective of those in the tissues. As a result, they have great potential as accurate diagnostic and prognostic markers, as well as being viable therapeutic targets for treating diabetes complications. Not only do miRNAs inhibit translation by binding to the 3' untranslated region (3'UTR) of their target mRNA [24], they can also trigger gene activation [25,26]. There is an emerging interest in these small, evolutionarily conserved regulatory markers, the non-coding RNAs and their involvement in health and disease, and there is already strong evidence for their important roles in the developmental modulation of growth control, tissue architecture, signaling pathways and disease pathology regulation. However, in spite of the many miRNA studies performed thus far, their organization within the genome, as well as their regulatory biology, remain to be well defined. A large number of miRNAs has already been discovered and described in relation to their vast targets, and of these, a number of specific miRNAs have appeared as major regulators of particular aspects of disease pathologies including diabetes complications. A great deal of attention has recently been given to the role of miRNAs and the different diabetes complications. Therefore, this review will focus on the relevance of miRNAs in the macrovascular and microvascular defects in diabetes, with particular emphasis on the different phases of diabetic skin wound healing.

### 2.1. Macrovascular Complications

#### 2.1.1. Cardiomyopathy

Diabetes is an independent risk factor and a major cause of chronic cardiovascular complications, some of which affect the vasculature, leading in particular to coronary arterial disease, stroke, hypertension, atherosclerosis and ultimately to heart failure, characterized by the presence of molecular, structural and functional changes [27,28]. Cardiomyopathy, a weakening of the heart muscle, is the measurable deterioration of the function of the myocardium. About 80% of deaths associated with diabetes are due to heart disease [29,30,31]. It is of paramount importance to identify early biomarkers to be able to diagnose the disease while in its early stages and through this alter the prognosis. Development of novel interventions for therapy is also needed. Several studies have focused on identifying miRNAs, as diagnostic and therapeutic tools involved in the symptoms and development of diabetic cardiovascular complications. Several reviews have recently been published summarizing important findings, regarding the putative mechanisms of the disease in relation to the miRNAs involved for use as potential diagnostic markers, as well as therapeutic targets [29,32,33,34,35,36,37,38,39]. One study identified four miRNAs in circulation, comprising miR-1, miR-16, miR-26a, and miR-133a, the latter, a possible biomarker that can help distinguish Takotsubo cardiomyopathy from ST-segment elevation acute myocardial infarction [40]. These pathologies have until now been clinically indistinguishable. Furthermore, the downregulation of miR-548 family members (miR-548c and -548i), was identified through genome-wide miRNA-microarray using peripheral blood mononuclear cells, which are more easily available than heart tissue samples [41]. This suggested that these markers could be used to detect early heart failure [41]. Moreover, miR-22, expressed in cardiac and skeletal muscle, was found upregulated during myocyte differentiation and cardiomyocyte hypertrophy, and it has been suggested as a regulator of cardiomyocyte hypertrophy and cardiac remodeling, with Sirt1 and Hdac4 as its targets [42]. Left ventricular hypertrophy is a compensatory mechanism in response to cardiac stress leading to heart failure. Recently, a study found that cardiac fibroblasts secrete miRNA-enriched exosomes. They identified miR-21-3p (miR-21), derived from fibroblast exosomes, as a potent paracrine-acting RNA molecule that induces cardiomyocyte hypertrophy [43]. In addition, pharmacological inhibition of this biomarker could lead to attenuation of the pathology [43].

#### 2.1.2. Atherosclerosis

Hypertension and hyperlipidemia significantly contribute to the formation and progress of the atherosclerotic plaque [44,45]. In addition, both endothelial dysfunction, induced by high circulating glucose and lipid levels, and inflammation mostly provoked by immune responses mediated by macrophages and T-cells, has been implicated in the pathogenesis of atherosclerosis and vascular disease [46,47]. These processes are highly regulated by miRNAs and specific miRNAs have now been shown to play crucial roles in regulating lipid metabolism [48] and inflammation [47]. Several reviews have documented this topic, from oxidative stress [49,50] inflammation [51,52] and the development of atherosclerotic plaque [53]. The use of miRNAs as biomarkers for atherosclerosis diagnosis and prognosis [54,55] and their relation to aging [56] has been reviewed recently. Furthermore, miR-155 has recently been implicated with atherosclerosis, as a modulator of actin cytoskeleton organization in endothelial cells, through alterations in the small GTPase RhoA and myosin light chain kinase [57]. In addition, inhibition of miR-155 with antagomiR-155 can decrease lipid-loading in macrophages and reduce atherosclerotic plaques [58]. MiR-144-3p has also been implicated as essential for the regulation of both cholesterol homeostasis and inflammatory reactions [59]. Furthermore, miR-21 has been identified as having a protective role in cardiomyocyte apoptosis induced by ischemia-reperfusion and hypoxia-reperfusion, dependent on the phosphatase and tensin homolog (PTEN) and the serine/threonine-specific protein kinase AKT/PKB pathway [60]. Platelets play an important role in atherosclerosis and platelet-released miR-223 working via the insulin-like growth factor (IGF)-1 receptor can promote vascular endothelial cell (VEC) apoptosis induced by advanced glycation end products (AGE) [61]. This indicates that platelets can modulate VEC apoptosis through the release of miR-223. In addition, the nuclear receptor, liver X receptor (LXR) signaling in macrophages can modulate cholesterol homeostasis and the inflammatory response, pathways involved in atherosclerosis. Moreover, recent work identified miR-206 as a putative regulator of LXRα in macrophages, with inflammatory stimuli greatly inducing miR-206 expression [62]. Resistin, an insulin resistance biomarker, seems to be a key factor in atherosclerosis and as reported recently high glucose stress was able to induce a significant decrease in miR-492 expression, with a consequent upregulation of resistin expression [63]. On the other hand, upregulation of miR-492 attenuated endothelial cell migration and lipid accumulation.

### 2.2. Microvascular Complications

#### 2.2.1. Diabetic Retinopathy

Diabetic retinopathy is one of their leading causes of blindness [64]. It affects up to 80% of all patients who have had diabetes for 10 years or more [65,66]. Risk factors for progression of diabetic retinopathy are hyperglycemia, hypertension, hyperlipidemia, and smoking [65,66]. Hyperglycemia, insulin signaling abnormalities and inflammation lead to retinal microvascular defects, neuroretinal dysfunction and degeneration [32,38,67]. Major symptoms include pericyte death and thickening of the basement membrane, leading to weak vascular walls. miRNAs play an important role in the mechanisms underlying the pathogenesis of retinopathy [38,68]. A summary of the important miRNAs for retinopathy is presented in Table 1. One of the first studies demonstrating the importance of miRNAs in diabetic retinopathy carried out a large miRNA-expression profiling assay on the retina and retinal endothelial cells of streptozotocin-induced diabetic rats [69]. Kovacs and colleagues found several miRNA signatures for the upregulation of the transcription factor nuclear factor (NF)-kB, vascular endothelial growth factor (VEGF), and p53, reflecting the pathologic alterations of retinopathy [69]. Moreover, they found that, in particular, miR-146 is a potential therapeutic target through its inhibition of NF-kB activation in retinal endothelial cells [69]. They also indicated that the miR-34 family is upregulated in diabetic rats and some of them are important markers in the retina. Another study indicated the involvement of miR-34a in the proliferation and migration of retinal pigment epithelial (RPE) cells, important in vitreoretinopathy [70]. In addition, apoptosis of retinal neurons is one of the important players in diabetic retinopathy [71,72]. Studies in streptozotocin diabetic rats have suggested that miR-29b may have a protective role against the apoptosis of retinal ganglion cells and cells of the inner nuclear layer of retinas [73]. Another group, using microarray screening assays, found that 304 miRNAs were differentially expressed in the transforming growth factor (TGF)-β2-induced epithelial-mesenchymal transition of human retinal pigment epithelium cells [74]. An important finding because this event is imperative during the development of proliferative vitreoretinopathy. Moreover, not only can TGF-β2 mediate fibrosis but it can also induce cell migration [75] and therefore it would be important to identify the targets for these miRNAs. One of the key mechanisms causing retinal microvascular injury in diabetes is endothelial cell damage. Recent results have identified a novel mechanism by which miR-195 regulates sirtuin (SIRT)-1 mediated tissue damage in diabetic retinopathy. The expression of miR-195 was found to be upregulated in retinas of diabetic rats while intravitreal injection of antagomiR-195 ameliorated levels of SIRT-1 [76]. Hyperglycemia has been suggested as the cause for high miR-195 expression levels found [76]. Furthermore, hypoxia-inducible factor 1 alpha (HIF1α) and VEGF are both implicated in the pathogenesis of diabetic retinopathy [77]. Ling and colleagues recently found that there is a cross-talk between HIF1α and VEGF through the expression of common miRNAs, such as miR-106a, and that silencing either HIF1α or VEGF increased the availabilities of the shared miRNAs [78]. In addition, it has been shown that miR-126 is not only downregulated under hypoxic conditions *in vivo* and *in vitro*, but it can also modulate the expression of VEGF and MMP-9 protein levels in monkey chorioretinal vessel endothelial cells (RF/6A) [79]. Moreover, miR-200b has been involved in the regulation of oxidation resistance (Oxr)-1, a protein that controls the sensitivity of neuronal cells to oxidative stress, in the retinas of Akita mice, a model of type 1 diabetes, where it appears to be upregulated [80], while in another study the same miRNA has been found downregulated upon high glycemia in diabetic retinas and endothelial cells, having VEGF as a possible target [81]. These findings could be very significant from a therapeutic perspective, since miRNAs are important in neovascularization, matrix protein accumulation and vascular permeability, all important contributors for loss of vision. Much more work *in vivo* needs to be done in order to identify and validate the specific targets and pathways that can be modulated by some of these differentially expressed biomarkers.

**Table 1:** Summary of miRNAs that are involved in diabetic retinopathy.

microRNAs	Diabetic Retinopathy—miRNA Functions
miR-132, miR-155, miR-146, miR-21	Upregulated with increased NF-kB, ICAM-1 and MCP-1, in diabetic retinal endothelial cells and retinas [69].
miR-34 family	Upregulated in diabetic rats upon VEGF and p53 responses, including in retinas [69].
miR-34a	Downregulated in subconfluent retinal pigment epithelial cells. It can inhibit their proliferation and migration [70].
miR-29b	Upregulation at the early stages of diabetes with potential target the cellular activator of x cellular activator of PKR, RAX (PKR activator X), in retinal ganglion cells [73].
miR-195	Upregulated in retinas of diabetic rats. Regulates sirtuin 1 mediated tissue damage, in human retinal and dermal microvascular endothelial cells [76].
miR-195	Upregulated in retinas of diabetic rats. Regulates sirtuin 1 mediated tissue damage, in human retinal and dermal microvascular endothelial cells [76].
miR-200b	Downregulated upon high glycemia with VEGF as a direct target, in diabetic retinas and endothelial cells [81]. Upregulated in Akita mouse retinas. Regulates the expression of oxidation resistance-1 [80].
miR-126	Downregulated by hypoxia and reduced in the retinal tissue of streptozotocin-induced diabetic rats. VEGF and MMP-9 are possible targets [79].

NF-kB, Nuclear factor kappa-light-chain-enhancer of activated B cells; ICAM-1, Intercellular adhesion molecule 1; MCP-1, Monocyte chemoattractant protein-1; VEGF, Vascular endothelial growth factor; RAX, PKR activator X; HIF1α, Hypoxia-inducible factor 1-alpha; Oxr-1, Oxidation resistance-1; MMP-9, Matrix metallopeptidase 9.

#### 2.2.2. Diabetic Nephropathy

Diabetic nephropathy, occurs in about 50% of the patients with T2DM, resulting in chronic kidney disease and organ failure [82,83]. It is the leading cause of progressive kidney disease and it contributes to the increased morbidity and mortality among individuals with diabetes [84,85]. Both high blood pressure and high levels of blood glucose increase the risk for nephropathy. Moreover, as kidney filtration starts to fail, various proteins start leaking into the urine causing proteinuria, and wastes, such as uric acid, accumulate in the blood. As a consequence osmolality increases and blood pressure climbs, enhancing kidney failure [86,87]. Chronic inflammation [88,89] and oxidative stress [90,91] also contribute to this pathology [32]. One of the most important players in the progression of renal diseases is TGF-β. However, additional downstream targets and pathways remain to be identified [92,93]. miRNAs have been identified as regulating their own gene expression, as well as influence entire signaling networks [19]. Much work has been dedicated into trying to understand how miRNAs regulate and are regulated by factors that contribute to kidney disease [32,33,94]. Nephropathy is one of the research areas where most miRNA dysregulations have been identified. Recent reviews have analyzed the effects of miRNAs, on diabetic nephropathy, from normal kidney function [95,96], to kidney fibrosis [97], glomerular podocyte dysfunction [98], blood pressure regulation [99], the renin-angiotensin system, advanced glycation end products (AGE)/RAGE (receptor of AGEs) signaling under oxidative stress [100] and kidney inflammation [32]. Most recently, the TGF-β signaling pathway has been implicated with upregulating miR-21 and miR-192, whereas downregulating miR-29 and miR-200, important in renal fibrosis [101]. Mesangial proliferation and glomerular hypertrophy are abnormalities in nephropathy. Results have indicated that down-regulation of miR-34a can inhibit mouse mesangial cell proliferation and, therefore, alleviate glomerular hypertrophy *in vivo* [102]. In addition, regulation of histone deacetylase (HDAC) actions and nephrin acetylation by miR-29 could contribute to podocyte homeostasis and renal function [103]. The study demonstrated that overexpression of miR-29a attenuated HDAC4 signaling, nephrin ubiquitination, and urinary nephrin excretion, associated with diabetes and restored nephrin acetylation, demonstrating a possible protective effect of miR-29a against diabetic kidneys. Moreover, the use of miRNAs as biomarkers for nephropathy, both diagnosis and follow-up [67,85] and as potential therapeutic targets [104], has been further reviewed elsewhere.

#### 2.2.3. Diabetic Neuropathy

Diabetic neuropathy is nerve damage occurring in the presence of diabetes, with prevalence greater than 50% in patients with long-standing disease [105,106,107]. There are sensorimotor and autonomic neuropathies. Sensorimotor neuropathy may include pain, paraesthesia and sensory loss [108,109], while autonomic neuropathy may contribute to myocardial infarction, malignant arrhythmia and sudden death [110]. It is chronic hyperglycemia and associated metabolic defects (mainly oxidative stress, vascular damage and ischemia) that can lead to injury of nerve fibers throughout the body [32,109,111,112]. Sensory neurons are responsible for diabetic peripheral neuropathy, often resulting in pain or numbness in hands and feet [108,109]. Neuropeptide expression and respective signaling pathways, responsible for inducing pro- or anti-inflammatory cytokine expression, are highly involved in diabetic wound healing. However, they are compromised in the neuropathic state, as neuropeptide release through the C-nociceptive fibers is impaired [13,113,114,115]. Unlike for nephropathy and heart disease, little is known about the role of miRNA in neuropathic complications. However, a recent study using genome wide studies and miRNA sequencing was able to identify three miRNAs that were differentially regulated in neuropathy: miR-30d-5p and miR-125b-5p, two major players in regulating the expression of TNF-α, brain-derived neurotrophic factor (BDNF) and signal transducer and activator of transcription (STAT)-3, as well as miR-379-5p, all closely associated with neuropathic pain [116]. Furthermore, miR-203, miR-96 and miR-7a have also been identified as regulators of protein expression in the dorsal root ganglion, also associated with neuropathic pain [117,118,119]. In addition, polymorphisms have been identified in miR-128a and miR-146a, with susceptibility for diabetic polyneuropathy, and in miR-146a and miR-27a, with susceptibility for cardiovascular autonomic neuropathy [120]. Regarding nerve repair, in particular Schwann cell migration, a recent study identified miR-9 as an important regulator [121]. Even though, potential miRNAs have been identified as regulators of some particular neuropathies, much remains to be done in order to further validate their role in the mechanisms underlying these neuropathies.

## 3. MiRNA in Diabetic Wound-Healing Impairment

**Figure 1:**
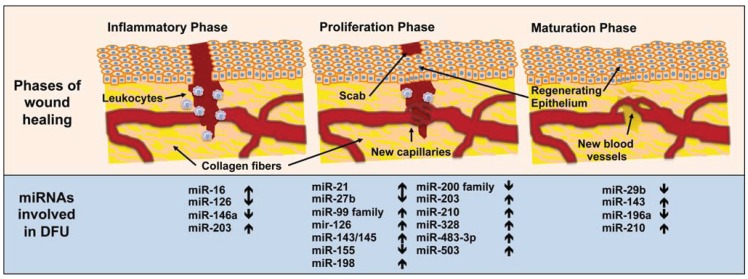
Schematic representation of the different wound healing phases and a summary of the most relevant miRNAs thus far identified, that are involved in wound healing impairment in diabetes. Arrows indicate wound up- or downregulation.

One of the major causes of diabetes-associated morbidity and mortality is lower limb amputation, which is a consequence of DFU [3,13]. In contrast to acute wounds that progress through the phases of wound healing linearly in healthy individuals, chronic wounds in diabetic patients become stalled in different phases and progression does not occur in synchrony due to diabetes associated neuropathy, microangiopathy and impaired immune function [122]. Recent studies have demonstrated the importance of miRNAs in the regulation of gene expression in various cells of the skin, including stem cells, immune cells and keratinocytes. In mesenchymal stem cells (MSCs), miR-27b was identified as a unique signature of the stem cell niche in burned mouse skin, suppressing the migration of mMSCs by targeting stromal cell-derived factor (SDF)-1D [123]. In addition, miR-27b was shown to rescue impaired bone marrow-derived angiogenic cell angiogenesis via thrombospondin (TSP)-1 suppression [124]. It has been shown that keratinocytes recognize invading pathogens by various receptors, among them Toll-like receptors (TLRs). Recent results show that Toll-like receptors may be able to alter the miRNA expression profile of keratinocytes, including miR-146a [125]. This could implicate the role of miRNAs in modulating the innate immune response of these cells. Some of these studies have been extensively reviewed [126,127], therefore, we will focus our attention on the important roles of miRNAs in the different phases of wound healing, as summarized in Figure 1.

### 3.1. Inflammatory Phase

Immediately after the skin barrier is breached, coagulation is triggered and usually homeostasis is achieved within minutes. Independently of the extent of the wound, before tissue integrity is restored, pathogens can infect the exposed tissues underlying the damaged skin. This is when the inflammatory phase of wound healing begins (Figure 1). During this period the immune system identifies and eliminates the infecting pathogens and damaged cells. To do so, the various immune cells travel to the wound site and interact with the various skin cells, in an orchestrated orderly fashion [128,129,130]. Selective leukocyte homing to the wound site is controlled either by the inflamed tissue, through the regulation of chemokine gradients [131] or by the infectious agents themselves, mainly by N-formil-methionine peptides, with bacterial origin [132], making the immune response proportional to the wound area. We are now starting to unravel how miRNAs are involved in the regulation of both chemokine and chemokine receptor gene expression.

Zhai and others revealed that the expression of important inflammatory chemokines, like chemokine (C-C motif) ligand (CCL)2, important for monocyte, basophil, dendritic cell and Th2 T-cell chemotaxis, is controlled by miRNAs [133]. Nakamachi and others later identified miR-124a as responsible for the diminished CCL2 mRNA stability and decreased CCL2 expression in fibroblast-like synoviocytes from patients [134]. Dorhoi and others concluded that miR-223 directly targets chemokine (C-X-C motif) ligand (CXCL)2 and CCL3, both important for neutrophil recruitment to the wound site [135]. This data is of particular importance for the understanding of the diabetic wound healing impairment, since, in a diabetic rabbit model, both CXCR1 and CXCR2, the receptor for CXCL2, were found to be under-expressed in endothelial cells, surrounding the wound, when compared to controls [136]. Moreover, IL-8, the ligand for CXCR1, was also found to be under-expressed in endothelial cells from DFUs [137], and its expression is regulated by miR-155 (upregulated) [138] and miR-93 (downregulated) [139]. It is not clear yet if any of these miRNAs are involved in modulating the DFU pathology.

Leukocyte retention is also important for the destruction of affected cells and tissue repair [140]. Harris and others [141] have demonstrated that miR-126 decreases leukocyte adherence to endothelial cells, and Ortega and others [142] have demonstrated that it regulates vascular cell adhesion molecule (VCAM)-1 expression. T2DM patients have decreased levels of this particular miRNA [143]. Moreover, the expression of CD38, a glycoprotein necessary for immune cell adherence to human vein endothelial cells [144], is downregulated by miR-140-3p, that specifically binds to CD38 3'-UTR [145]. T2DM patients also have increased levels of miR-140-3p [146], but there is no data on CD38 activation and expression in these patients.

Inflammation is also directly or indirectly controlled by miRNAs. Figure 1 indicates some of the miRNAs involved in this process in regard to wound healing. miR-16 has been shown to inhibit cyclooxygenase (COX)-2 (also known as prostaglandin-endoperoxide synthase 2) expression [147], demonstrating a systemic anti-inflammatory effect. COX-2 is overexpressed in T2DM patients [148], but it is not yet clear if this effect is due to miR-16 modulation. Nevertheless, it is known that there is a link between miR-16 and obesity [149], one of the major pathologies leading to T2DM. Interestingly, in humans, surgery-induced weight loss led to a decrease in miR-16 levels, but the same was not true for diet-induced weight loss [150]. On the other hand, miR-203, usually associated with β-cell apoptosis in type 1 diabetes mellitus (T1DM) [151], is also responsible for the inhibition of TNF-α and IL-24 expression in the skin [152]. Since increased TNF-α levels favor DFU formation [153], the overexpression of miR-203 should give some degree of protection from DFU formation to these patients. Since DFU formation usually occurs many years after β-cell depletion, it would be important to know if miR-203 levels stay high after β-cell depletion, conferring protection from DFU or if miR-203 levels decrease.

After the pathogen burden has been eliminated, inflammation has to be terminated, in order for the healing to continue [109]. This is one of the most important steps where diabetic wound healing fails [153]. In fact, one of the common denominators for all chronic wounds is the persistent inflammatory state [154]. One particular miRNA, that plays a key role as a molecular brake on inflammation, miR-146a, is significantly downregulated in diabetic mouse wounds [155]. Although DFU patients are unable to mount an acute and sufficient immune response to wound infecting bacteria, they present a systemic pro-inflammatory environment, with elevated IL-1, TNF-α, IL-6 and regulated on activation, normal T-cell expressed, and secreted (RANTES) levels [88]. Hyperglycemia alone is able to activate the transcription factor NF-kB [156], impairing leukocyte activation [157] and migration [158]. It is reasonable to speculate that the downregulation of miR-146a is linked to, or even responsible for all these effects, as proposed by Balasubramanyam and others [159]. Their results have demonstrated an inverse correlation between miR-146a expression and glycated hemoglobin, insulin resistance, tumor necrosis factor receptor-associated factor (TRAF)-6, and NF-kB mRNA levels and circulatory levels of TNF-α and IL-6.

### 3.2. Proliferation Phase

Cytokines and chemokines released by inflammatory cells eventually attract fibroblasts and myofibroblasts to the injury site, initiating the proliferation phase, characterized by the formation of granulation tissue and deposition of collagen and glycosaminoglycans [160]. In fact, cytokines control the expression of various miRNAs in fibroblasts [161]. One such miRNA is miR-155, of which transfection, in a mouse model of lung fibrosis, increased fibroblast migration and, consequently, lung fibrosis [161]. A recent study [162] has shown that miR-155 expression is significantly decreased in blood mononuclear cells from T2DM patients. The expression of miR-155 is also decreased in diabetic mice and its over-expression is able to prevent cardiac fibrosis in these mice [163]. Madhyastha and others [164] have analyzed the miRNA signature in diabetic wound healing and identified 14 miRNAs that were differentially expressed in diabetic skin, among these, miR-21 showed increased expression in diabetic skin, but decreased expression during diabetic wound healing (Figure 1). They also demonstrated that reduced miR-21 expression in diabetic wounds decreases the rate of fibroblast migration [164].

With the onset of the proliferation phase new blood vessels are created to supply the area undergoing regeneration with nutrients and oxygen, and extracellular matrix (ECM) is synthesized in order to rebuild the damaged tissue. During this phase the injured dermis starts getting red (erythema) and gain volume (edema) [165]. As macrophages and other cells produce and release VEGF, they play a direct role in neovascularization and angiogenesis [166]. Various miRNAs involved in the regulation of angiogenesis appear to be dysregulated in diabetic patients [167]. miR-27b is downregulated in endothelial progenitor cells (EPCs) from T2DM patients [168]. In diabetic mice, its overexpression rescued impaired bone-marrow derived angiogenic cell function via TSP-1 suppression, and topic miR-27b delivery improved diabetic mouse skin wound closure [164]. In a rat model, miR-328 has been shown to inhibit the formation of capillary structures by targeting CD44 expression [169], and others have shown that miR-328 is overexpressed in diabetic individuals [170]. miR-503 has also been shown to impair angiogenesis and its expression is upregulated in T2DM patients [171]. Furthermore, the miR-143/145 cluster has been implicated in insulin resistance and the development of T2DM [172,173] and has also been demonstrated to inhibit angiotensin II formation [174], by targeting angiotensin-converting enzyme (ACE) thus, impairing wound healing (Figure 1). On the other hand, ACE inhibitors have been shown to reduce all-cause mortality in diabetic patients [30], leading us to conclude that the miR-143/145 cluster may also have a systemic protection effect in diabetic patients. Moreover, miR-126 also promotes endothelial cell proliferation, migration and angiogenesis [175,176] and is significantly reduced in susceptible individuals and T2DM patients [143]. Aberrant expression of miR-16, miR-20a, miR-21, miR-106a, miR-130a and miR-203, have been found in venous ulcers studies [177]. These miRNAs have been predicted to target multiple genes important for wound healing, like early growth response factor 3, vinculin and the leptin receptor [177].

The migration, proliferation and differentiation of keratinocytes are, altogether, a key step in wound healing because hair follicle keratinocytes (first) and epidermal keratinocytes (later) will eventually fill in the gap created by the wound and restore the integrity of the skin. Various miRNAs, such as miR-198, miR-203 and miR-483-3p, are known to inhibit keratinocyte migration and proliferation [178,179,180]. All these miRNAs are downregulated in normal skin wounds, whereas the expression of miR-198 persists in diabetic wounds [178], and the expression of both miR-203 and miR-483-3p is upregulated in diabetic mice [151,181]. On the other hand, miR-95, miR-203 and miR-210 promote keratinocyte differentiation [182] and both miR-203 and miR-210 are upregulated in diabetic mice [151] and miR-210 is upregulated in both T1DM [183] and T2DM [184] patients. Moreover, miR-21 has a variety of effects on wound healing, one of which is to promote keratinocyte migration and re-epithelialization [185,186]. The expression of miR-21 is usually increased in diabetic patients, because it is overexpressed in response to high glucose levels [187], but is decreased in diabetic wounds, when compared to normal wounds [164]. The expression of miR-21 must be tightly controlled in skin wounds because despite its beneficial effect on wound healing, its overexpression inhibited epithelialization and granulation tissue formation in a rat wound model [177]. It has also been demonstrated that the miR-99 family members, overexpressed in diabetic patients [188], reduce re-epithelialization of dermal wounds by targeting the AKT/PKB/mTOR signaling pathway [189]. Furthermore, the miR-200 family of miRNAs appears to controls epithelial to mesenchymal transition (EMT) [190], an essential phenomenon for wound re-epithelialization to occur, and is dysregulated in diabetic mice [191].

### 3.3. Maturation Phase

The maturation phase can last several weeks and occurs once the wound has closed (Figure 1). It is characterized by ECM adjustment, remodeling of collagen from type III to type I, and the replacement of granulation tissue by scar tissue [3]. Cellular activity decreases and the number of blood vessels in the wound decrease [192]. Results indicate that mRNA expression for α1-procollagen was reduced in diabetic wounds in a diabetic mouse model, resulting in increased matrix rigidity [193]. In the same study reduced alpha-smooth muscle actin (α-SMA) staining in the cells and lack of orientation of fibroblasts in diabetic skin, was observed [193], which could affect the efficient contraction of the wound [194]. Interestingly, miR-196a expression is down-regulated by TGF-β that, in turn, is increased in diabetes [195]. Moreover, miR-196a has been shown to down-regulate the expression of type I and III collagens in fibroblasts [196], improving wound healing in a keloid fibroblast model. Recent studies have also demonstrated that miR-196a inhibits the expression of the homeotic gene Hoxc8, a repressor of brown adipogenesis [197]. They have shown that forced expression of miR-196a in mouse adipose tissue increases energy expenditure, thereby rendering the animals resistant to obesity and diabetes. These observations suggest that the overexpression of miR-196a in diabetic patients may also improve wound healing.

Activin A has an important impact in the maturation phase of wound healing, as it promotes the replacement of fatty tissue by connective tissue [198], as well as, re-epithelialization and granulation tissue formation [199]. The high levels of activin A, observed in blood of T2DM patients [200] inhibits insulin action in cardiomyocytes via the induction of miR-143, which in turn, suppresses the novel regulator of insulin action, the oxysterol-binding protein-related protein 8 [173]. On the other hand, the expression of miR-210 is enhanced by high glucose in endothelial cells cultured *in vitro* [184]. It has also been found upregulated in diabetic mice [151] and diabetic patients [183,184], silencing activin A receptor type 1B [201], possibly compensating the effect of the high activin A levels. miR-29b has been demonstrated to directly target ECM genes, such as fibronectin, collagen type I, and collagen type III [202,203]. The *in vivo* topical application of miR-29b to mouse wounds improved collagen type III/I ratios and generated a significantly higher matrix metalloproteinase 8 activity, enhancing scarless wound healing [204]. As stated above, repairing a defect in the human skin is a highly orchestrated physiological process involving numerous factors that act in synergy to re-establish barrier function by regenerating new skin. It has been shown by several studies that miRNAs are very important markers and modulators of the different phases of wound healing [205,206,207]. In this section we have analyzed the involvement of miRNAs in the dysregulation of diabetic wound healing and summarized the phases of wound healing and the miRNAs involved in DFU in Figure 1 and Table 2.

**Table 2:** Summary of the most relevant miRNAs involved in the different phases of wound healing.

Phases of Wound Healing	miRNA Involved	Functions
**Inflammation**	miR-16	Inhibits COX-2 expression in monocytes [147].
	miR-126	Decreases leukocyte adherence to endothelial cells [141].
	miR-146a	Key role as a molecular brake on inflammation [155].
	miR-203	Inhibits TNF-α and IL24 expression [152].
**Proliferation**	miR-21	Promotes keratinocyte migration and re-epithelialization [185,186], increases the rate of fibroblasts migration towards the wound [164] and delays epithelialization [177].
	miR-27b	Rescues impaired BMAC angiogenesis via TSP-1 suppression [124].
	miR-99 family	Reduces re-epithelialization of dermal wounds [189].
	mir-126	Promotes endothelial cell proliferation, migration and angiogenesis [175,176].
	miR-143/145	Inhibits angiotensin II formation [174].
	miR-155	Inhibits KGF expression in fibroblasts [161].
	miR-198	Inhibits keratinocyte migration [178].
	miR-200 family	Controls epithelial-mesenchymal transition [190].
	miR-203	Inhibits keratinocyte proliferation and migration [180] but promotes keratinocyte differentiation [182].
	miR-210	Promotes keratinocyte differentiation [182] and silences Activin A receptor type 1B [201].
	miR-328	Inhibits the formation of capillary structures [169].
	miR-483-3p	Inhibits keratinocyte proliferation and migration [179].
	miR-503	Impairs angiogenesis [171].
**Maturation**	miR-29b	*In vivo* topical application to mouse wounds improves collagen type III/I ratios and generates a higher matrix metalloproteinase 8 activity [204].
	miR-143	Inhibits insulin action in cardiomyocytes from T2DM patients [200].
	miR-196a	Decreases expression of type I and III collagens in fibroblasts [196] and its overexpression renders mice resistant to obesity and diabetes.
	miR-210	Silences activin A receptor type 1B [201].

COX-2, cyclooxygenase-2; TNF-α, Tumor necrosis factor α; IL24, Interleukin 24; BMAC, bone marrow-derived angiogenic cell; TSP-1, thrombospondin-1; KGF, KGF expression in fibroblasts.

## 4. Potential of MiRNAs as Early Biomarkers for Detection and Treatment of Diabetic Foot Ulceration

The ideal biomarker for any disease would be a molecule that would be produced in the earliest stages of a particular disease and that would not be produced, at least in the same quantities, in any other condition. Such molecule should be easily collected by non-evasive means, preferably from the blood or urine, and would be stable enough to be quantified in conventional clinical laboratories. Although this perfect biomarker is still elusive, in the case of diabetes and its complications, the conventional biomarkers are either not specific, such as cholesterol, creatinine, free fatty acids, lipoproteins, c-reactive protein or adipokines, or not able to identify the early stages of the disease, like hyperglycemia, insulin, glutamate decarboxylase, islet-cell dysfunction and auto-antibodies in the case of T1DM, tyrosine phosphatases or incretin levels. Moreover, we are still not able to easily identify individuals at risk of developing diabetes mellitus and its associated complications. Ideally, a biomarker should also change in form or quantity with disease progression or/and with a therapeutic intervention. None of the referred biomarkers or any other currently used by clinicians meets all or even most of the referred specifications. This is why miRNAs hold a promising potential. miRNAs are easily collected and stable enough to be analyzed in a clinical environment, possibly using automated methods [208,209,210,211]. The use of miRNAs as biomarkers for the various types of diabetes, as well as their macrovascular and microvascular complications has been recently and extendedly reviewed [36,67,94,212,213,214] and will not be discussed here. Instead, we will focus on the use of miRNAs as biomarkers and therapeutic targets for the diabetic wound healing complications.

### 4.1. MiRNAs as Biomarkers for the Development of Chronic Diabetic Ulceration

The idea of using miRNAs as biomarkers for diabetes and its complications has come from studies using large cohorts of patients, usually using microarray profiling, later confirmed by qPCR, where several miRNAs have been identified as being dysregulated in the blood, either whole blood or its fractions, from diabetic patients. These studies have even allowed for the discrimination of a specific miRNA profile for T1DM [183,215,216] and T2DM [142,217]. Despite the large amount of data collected, no specific miRNA has been identified as being a biomarker for diabetic wound healing impairment. Nevertheless, the accumulating data on miRNA dysregulation in DFU, here reviewed, allows us to predict good candidates to be tested as possible DFU biomarkers (Figure 1 and Table 2).

The reduced expression of miR-21 in diabetic wounds [164] and the multitude of its functions exerted in wound healing [186] makes it a good candidate as a DFU biomarker. miR-21 is involved in the various phases of wound healing, especially in the control of keratinocyte and fibroblast migration to the wound site [164,185]. The reduced expression of miR-21 is able, by itself, to significantly delay wound re-epithelization [177].

Another good candidate would be miR-126. Its expression is significantly reduced in susceptible individuals and T2DM patients. miR-126 is involved in leukocyte migration to the wound site [141,142], endothelial cell proliferation, migration and angiogenesis [175,176]. The fact that miR-126 is decreased in individuals susceptible for diabetes [143], and the lack of evidence indicating that it is decreased in other non-related pathologies, makes it a promising marker for early diabetes detection.

Both miR-203 and miR-210 are also good biomarker candidates because of their increased expression in diabetic mouse models [151] and in both T1DM and T2DM [183,184]. These miRNAs have been predicted to target multiple genes important for wound healing [177]. miR-203 is associated with β-cell apoptosis [151] and it also inhibits the expression of TNF-α and IL-24 in the skin [152]. In addition, it inhibits keratinocyte migration and proliferation [178,179,180], but promotes keratinocyte differentiation [182]. miR-203 has also been associated with neuropathic pain [117,118,119]. On the other hand, miR-210, whose expression is enhanced by hypoxia and high glucose levels [184], silences activin A receptor type 1B [201] and, similarly to miR-203, it also promotes keratinocyte differentiation [182].

### 4.2. MiRNAs as Therapeutic Targets for Chronic Diabetic Ulceration

Due to our lack of understanding on why wound healing is impaired in diabetes, the approach to DFU healing has relied on lower limb amputation or it is mainly addressed at the prevention level [218]. The therapeutic approach has been limited to the administration of growth factors [219] and, more recently, tissue reconstruction using endothelial progenitor cells [220], or stem cells [221], with limited results. miRNA-based therapy strategies show great potential, aided by the increasing rise of nanotechnology, with promising results and few side-effects [222].

The selective knockout of specific miRNAs, either by gene manipulation or by the use of antagomiRs has proven successful, both *in vitro* and *in vivo* [223,224]. Several miRNAs enhanced in diabetes or its complications have been targeted with relative success in diabetic mouse models, while others have been proposed as potential therapeutic targets [197]. miRNAs targeted with success in other pathologies or normal conditions may also prove useful in diabetes. For example, van Solingen and others have proven that miR-155 knockout mice display improved repair of dermal wounds [225]. Although the expression of miR-155 is not enhanced in diabetic patients, since it plays an important role in inflammation, it may be a tentative target to reduce the enhanced inflammation observed in DFU patients.

The replenishment of miRNAs is much more difficult due to several limitations, such as *in vivo* delivery methods and tissue specificity. However, once these limitations are overcome, they could also become a novel therapeutic strategy [119]. One other limitation for the use of miRNAs as therapeutic targets is their usually high redundancy. As an example, Kovacs and others [69] have proposed that miR-146 could be a good therapeutic target in diabetes, due to its role as a molecular brake on inflammation [159] and the fact that it is significantly downregulated in diabetic mouse wounds [155]. However, the upregulation of miR-146 in a mouse model, not only did it not downregulate several pro-inflammatory markers (IRAK-1 and TRAF-6) and cytokines (TNF-α, IL-6 and IL-1β) [226] but it also generated autoimmune disorders mimicking the human autoimmune lymphoproliferative syndrome [227]. In this case, the treatment with mesenchymal stem cells proved to be more efficient, because it not only upregulate miR-146a expression, but it also enhanced wound healing in diabetic mice [155].

Due to these difficulties, the use of miRNAs as therapeutic targets for DFU remains an open challenge and every diagnostic biomarker should be considered as a possible therapeutic target. For instance, the upregulation of both miR-21 and miR-126 should enhance wound healing by stimulating leukocyte migration to the wound site, promoting bacterial control and wound closure, through the enhancement of keratinocyte and fibroblast activation and migration and improved angiogenesis. The downregulation of miR-203 and miR-210 should also enhance wound healing by promoting keratinocyte migration and proliferation.

## 5. Conclusions

MiRNAs are a fascinating area of RNA biology due to their roles in the fine-tuning of many physiological processes, and their modulation in human diseases. miRNAs are regulatory molecules that contribute to numerous aspects and phases of diabetes and its complications, including the impaired wound healing observed in DFU. They can play diverse roles as activating specific signaling pathways, upregulating or downregulating the expression of certain genes, depending on the stimuli. It has become clear that some of these molecules may provide valuable information within a clinical setting, potentially acting as screening tools for high-risk patients, becoming early predictive diagnostic tools, while informing the treatment decision-making process. Many of the results obtained so far on several of these miRNAs, have been the result of large screening studies, or have been performed in *in vitro* cell systems. Therefore, many of these markers still need to be validated in *in vivo* settings, where the promiscuity and diversity of interactions may pose some serious problems and invalidate clinical applications. Most importantly, we do not yet understand how many of these miRNAs exert their functions, either because we lack critical knowledge of the pathways involved, or because we are only seeing a small fraction of the “big picture”, since most of these miRNAs exert different functions according to the tissues and conditions where they are expressed. Nevertheless, once validated *in vivo*, they may themselves be considered direct therapeutic targets.
